# Who Knew? Dopamine Transporter Activity Is Critical in Innate and Adaptive Immune Responses

**DOI:** 10.3390/cells12020269

**Published:** 2023-01-10

**Authors:** Adithya Gopinath, Phillip M. Mackie, Leah T. Phan, Rosa Mirabel, Aidan R. Smith, Emily Miller, Stephen Franks, Ohee Syed, Tabish Riaz, Brian K. Law, Nikhil Urs, Habibeh Khoshbouei

**Affiliations:** 1 Department of Neuroscience, University of Florida, Gainesville, FL 32611, USA; 2 Department of Pharmacology and Therapeutics, University of Florida, Gainesville, FL 32611, USA

**Keywords:** Dopamine transporter, immunity, innate immunity, adaptive immunity, B-cells, T-cells, dopamine, DAT

## Abstract

The dopamine transporter (DAT) regulates the dimension and duration of dopamine transmission. DAT expression, its trafficking, protein–protein interactions, and its activity are conventionally studied in the CNS and within the context of neurological diseases such as Parkinson’s Diseases and neuropsychiatric diseases such as drug addiction, attention deficit hyperactivity and autism. However, DAT is also expressed at the plasma membrane of peripheral immune cells such as monocytes, macrophages, T-cells, and B-cells. DAT activity via an autocrine/paracrine signaling loop regulates macrophage responses to immune stimulation. In a recent study, we identified an immunosuppressive function for DAT, where blockade of DAT activity enhanced LPS-mediated production of IL-6, TNF-α, and mitochondrial superoxide levels, demonstrating that DAT activity regulates macrophage immune responses. In the current study, we tested the hypothesis that in the DAT knockout mice, innate and adaptive immunity are perturbed. We found that genetic deletion of DAT (DAT^−/−^) results in an exaggerated baseline inflammatory phenotype in peripheral circulating myeloid cells. In peritoneal macrophages obtained from DAT^−/−^ mice, we identified increased MHC-II expression and exaggerated phagocytic response to LPS-induced immune stimulation, suppressed T-cell populations at baseline and following systemic endotoxemia and exaggerated memory B cell expansion. In DAT^−/−^ mice, norepinephrine and dopamine levels are increased in spleen and thymus, but not in circulating serum. These findings in conjunction with spleen hypoplasia, increased splenic myeloid cells, and elevated MHC-II expression, in DAT^−/−^ mice further support a critical role for DAT activity in peripheral immunity. While the current study is only focused on identifying the role of DAT in peripheral immunity, our data point to a much broader implication of DAT activity than previously thought. This study is dedicated to the memory of Dr. Marc Caron who has left an indelible mark in the dopamine transporter field.

## 1. Introduction

Dopamine transporter (DAT) regulates dopamine signaling in DAT-expressing cells in the CNS and in the periphery. Altered DAT activity is primarily implicated in neurological and neuropsychiatric diseases such as drug addiction, ADHD, autism and Parkinson disease (PD), but dysregulation of dopamine transmission is also associated with a host of peripheral functions, including regulation of blood pressure, insulin secretion and immune function [1,2,3,4,5,6,7,8]. In addition, the connection between psychostimulant-mediated modulation of peripheral immunity, DAT activity and peripheral dopamine levels in the patients affected by different psychotic disorders is well documented [1,6,7]. DAT activity, but not its expression, is significantly lower in resting lymphocytes of psychotic patients than healthy control subjects [6]. These data demonstrate that alterations in DAT activity in neuropsychiatric or neurological disorders occur in both periphery and CNS.

In a recent study, we presented evidence that functional DAT molecules are expressed at the plasma membrane of peripheral monocytes and macrophages where DAT activity, via an autocrine/paracrine signaling loop, regulates the macrophage response to immune stimulation [9]. A deeper inspection of the literature revealed that lymphocytes and monocytes express the catecholamine biosynthetic enzyme tyrosine hydroxylase, but not dopamine beta hydroxylase [10,11,12], indicating that immune cells produce dopamine [11,13,14,15] and store the synthesized dopamine in the VMAT2 positive vesicle [16]. While the mechanism of dopamine release from immune cells remains enigmatic, we have shown that LPS, an endotoxin, induces DAT-mediated dopamine efflux [9]. Functional characteristics of [^3^H]-dopamine uptake by lymphocytes [16] and monocyte-derived macrophages [9] revealed the canonical activity for DAT molecules on immune cells. Uptake and release of dopamine modulate both the releasing cell and neighboring cells, mediating a variety of functions, such as B-cell activation and production of immune mediators such as TGF-β and IL-10 [1,17,18,19]. It is unclear whether the dopamine that mediates these interactions is produced de novo or taken up from the surrounding environment via DAT, stored and released. Collectively, these data support the hypothesis that DAT activity regulates dopamine signaling in the microenvironment of immune cells, which in turn modulates peripheral immunity.

In addition to immune cells, DAT molecules are also expressed in the cells of peripheral organs such as thymus and spleen known to regulate innate immunity, adaptive immunity, and immune cell maturation [20,21,22,23] indicating a functional role for DAT in immune cells maturation and expansion. In rat thymocytes, the expression of dopamine-associated proteins such as DAT and VMAT2 is higher in CD8^+^ cells than CD4^+^ cells [24]. Our recent studies [9,25] support an immunosuppressive function for DAT, where blockade of DAT activity enhanced LPS-mediated production of IL-6, TNF-α, and mitochondrial superoxide levels, demonstrating that dopamine uptake and release modulate macrophage immune responses [9]. In the current study, we tested the hypothesis that in the DAT^−/−^ mice innate and adaptive immunity are perturbed. We found that genetic deletion of DAT results in an exaggerated baseline inflammatory phenotype in peripheral circulating myeloid cells. In peritoneal macrophages obtained from DAT^−/−^ mice, we identified increased MHC-II expression and exaggerated phagocytic response to LPS-induced immune stimulation, suppressed T-cell populations at baseline and following systemic immune stimulation and exaggerated memory B cell expansion. In DAT^−/−^ mice, norepinephrine and dopamine levels are increased in spleen and thymus, but not in circulating serum. In addition, DAT^−/−^ mice exhibited spleen hypoplasia accompanied with significant reduction in the spleen weight, increased splenic myeloid cells and exaggerated MHC-II expression further supporting a critical role for DAT in the peripheral innate and adaptive immunity, suggesting that DAT activity serves in an endogenous immune modulatory mechanism.

## 2. Methods

### 2.1. Study Design

To investigate whether immune cell subpopulations were altered in DAT^−/−^ mice, we tested four cohorts of mice under different conditions: WT + saline, WT + LPS (2 µg/g), DAT^−/−^ + saline, DAT^−/−^ + LPS (2 mg/g). PBMCs, peritoneal immune cells and splenocytes from all cohorts were analyzed via fluorescence minus one (FMO) analysis; see Supplemental Figures S1–S3. Sample sizes for mouse cohorts were determined by power analyses using preliminary data, with alpha = 0.05 and a power of 80% for group comparison. Sample preparation, data collection and data analysis were all performed by blinded investigators. Numbers of experimental replicates are described in figure legends as appropriate. All animal research was conducted in accordance with University of Florida IACUC, as described below.

### 2.2. Animals

Mice, WT C57/B6, males and females 6–9 months old, DAT^−/−^ on C57/B6 background, males, and females 6–9 months old, were used for experiments. LPS (2 µg/g) or saline were injected via the tail vein and animals were provided both hard pellets and water-softened food on the floor of the cage, as well as access to a water bottle and standard cage water supply. Animals were monitored from the time of injection to experimental endpoint four hours later. Euthanasia was performed via isoflurane overdose, followed by the procedures outlined below.

Animals were housed in the animal care facilities and maintained as approved by IACUC at the University of Florida, and followed guidelines established by National Institutes of Health. Food and water were available ad libitum in the home cage. The room was maintained under standard 12 h light/dark cycles, at 22–24 °C with 50–60% humidity.

### 2.3. Measurement of Tissue and Serum Monoamines and Their Metabolites Via HPLC

Wild-type (WT) and DAT^−/−^ mice were anesthetized, followed by whole blood collection via cardiac puncture. Whole blood (1 mL) collected, transferred to tubes containing Li-Heparin and PBS, centrifuged for 5 min at 1600 g to separate serum from other blood components. Serum was aliquoted and stored at −80 °C for monoamine and monoamine metabolites analysis. Immediately after blood collection, spleen and thymus were removed and snap-frozen in liquid Nitrogen, then placed in ice-cold 0.2 M HClO4 solution and sonicated at 30% power. The volume of 0.2 M HClO4 solution was 10 times the weight of each organ and 10 times the volume of Serum. The samples were subsequently centrifuged at 16,000× *g* for 15 min at 4 °C, the supernatants were placed in separate tubes, filtered, and the filtrates were placed in HPLC tubes. Monoamine and their metabolites were determined by HPLC analysis with electrochemical detection (HTEC, Amuza Inc, San Diego, CA, USA). Data were obtained from *n* = 4 mice per genotype.

### 2.4. LPS Administration

DAT^−/−^ (C57/B6 background) and WT (C57/B6) mice received 2 µg/g body weight LPS via tail vein injection. Briefly, WT and DAT^−/−^ animals were placed in a Broome restrainer, the tail vein identified/cleaned (70% ethanol) and an appropriate volume of 1 mg/mL LPS solution was injected in sterile saline (or saline vehicle control), for a final dose of 2 µg/g body weight, to induce systemic endotoxemia.

### 2.5. Blood Collection and PBMC Isolation

Materials, reagents, and equipment are detailed in Table 1, Table 2 and Table 3. Whole blood was obtained via cardiac puncture when the animal was deeply anesthetized via isoflurane. Up to 1 mL whole blood collected in a 1 mL syringe with a 25-guage needle via cardiac puncture, transferred to K2EDTA vacutainer tubes and held for up to 30 min prior to PBMC isolation. Whole blood was transferred from collection tube into a 5 mL FACS tube containing 1 mL sterile PBS (1:1 dilution in PBS) then overlaid atop 1 mL sterile Ficoll-Paque Plus (GE, 45-001-750) in 5 mL FACS tubes. Overlaid blood samples were centrifuged for 20 min at 400× *g* with brakes off and acceleration set to minimum. PBMCs collected from the interphase of Ficoll and PBS were transferred to a fresh 5 mL FACS tube (Table 1), suspended with 4 mL sterile PBS and centrifuged for 10 min at 100 g, and repeated once more. After the second wash, supernatants and cells were suspended in 200 uL PBS.

Whole blood (200 mL) was diluted in PBS containing Li-Heparin anticoagulant, centrifuged for 5 min at 2700× *g* to separate serum from other blood components. Serum was aliquoted and stored at −80 °C for cytokine analysis as described below.

### 2.6. Peritoneal Macrophage Harvest

As described in Ray et al. 2010 [26], DAT^−/−^ and WT control animal peritoneal macrophages were collected as follows. The abdomen was cleaned with 70% ethanol and wiped dry. Using forceps, the abdominal skin was tented, and a gentle incision was made to avoid damaging the peritoneal lining. 10 mL ice cold PBS was aspirated into a 10 mL syringe with a 1.5-inch 18 G needle, and with the needle placed at a 45-degree angle to the peritoneal membrane and inserted to the peritoneum with the bevel facing the midline. PBS was slowly injected, then slowly withdrawn over 1 min to obtain a thorough peritoneal lavage containing peritoneal macrophages and other resident immune cells. The cell suspension was centrifuged at 300× *g* for 5 min, supernatant aspirated and the pellet suspended in 100 mL sterile PBS and used for downstream analysis.

### 2.7. Spleen Dissociation

To extract splenocytes from each animal, spleens were removed and immediately placed into PBS. The organ capsule was subsequently disrupted between glass slides and rinsed with fluorescence-activated cell-sorter (FACS) buffer (PBS and 1% FBS), then punctured with an 18-gauge needle to release splenocytes. Splenocytes were collected by centrifugation at 4 °C and 380× *g* for 5 min, suspended in FACS buffer, then filtered through a 40 mm cell strainer. Splenocyte samples were then centrifuged again at 4 °C and 380× *g* for 5 min, and suspended in 70% isotonic Percoll solution, and underlaid beneath 4 mL 37% isotonic Percoll solution, and centrifuged for 30 min at 500× *g*, with brakes off and acceleration set to minimum. Splenocytes were harvested from the interphase between 70% and 37% Percoll. Cells were washed twice with sterile PBS, counted with trypan blue exclusion of dead cells, density adjusted and then 100 μL cell suspension (1 × 10^6^ cells/100 μL) aliquoted into 1.5 mL microcentrifuge tubes for staining in preparation for flow cytometry.

### 2.8. Flow Cytometry

Antibody concentrations, vendor, catalog numbers and fluorochromes are shown in Table 1. Reagent details are shown in Table 2. Immediately following counting and density adjustment, 100 μL cell suspension from each sample was distributed into microcentrifuge tubes for staining. PBMC staining with antibodies against CD11b, CD45, CD19, CD27, CD3, CD4, CD8, Ly6C, and Ly6G was performed on ice, protected from light, then cells were allowed to incubate for 30 min. Peritoneal macrophages were stained at room temperature with F4/80, CD11b and MHC-II, for 20 min. Splenocytes were stained at room temperature with F4/80, CD11b, MHC-II and CD19 for 20 min. Peritoneal macrophages and splenocyte staining were conducted at room temperature to ensure appropriate temperature for live cell phagocytosis as described below. All samples included viability dye to allow exclusion of dead cells from analysis.

**Table 1:** Reagents and Materials.

Reagent	Supplier	Catalog Number	Purpose	Concentration
Ficoll-Paque Plus	GE	45-001-750	PBMC isolation	N/A
LPS	Sigma		Immune stimulation	2 µg/g body weight
PBS	In house	N/A	PBMC isolation, FC	1×
K2EDTA Vacutainer	BD	366643	Blood collection	N/A
FACS tubes	Fisher		FC, mouse PBMC isolation	N/A
Fix/Perm Kit	eBioscience	88-8824-00	FC	Stock
Leucosep Tube	Grenier BioOne	227,290P	PBMC isolation	N/A
Syringe	Exel	26016	IP injection, cardiac puncture blood draw	N/A
Isoflurane	Patterson	07-893-8441	Anesthesia	1–5%
Phagocytosis beads	Sigma	L3280	Phagocytosis	0.5 mL
Legendplex	Biolegend	740150	Cytokine Analysis	N/A

**Table 2:** Antibodies.

Specificity	Clone/Species	Conjugate	Vendor	Catalog Number	Purpose	Dilution
CD11b	M170/Rat	PerCP-Cy5.5	Biolegend	101,228	FC	1:100
CD45	30-F11/Mouse	FITC	Biolegend	334,824	FC	1:200
CD19	6D5/Rat	BV605	Biolegend		FC	1:100
CD27	LG.3A10/Mouse	APC	Biolegend	124,212	FC	1:100
CD3	17A2/Mouse	PacBlue	Biolegend	100,214	FC	1:50
CD4	GK1.5/Mouse	AF700	Biolegend	100,429	FC	1:50
CD8a	QA17A07/Mouse	SV538	Biolegend	155,020	FC	1:50
Ly6C	HK1.4/Rat	BV785	Biolegend	128,041	FC	1:200
Ly6G	1A8/Rat	PE	Biolegend	127,607	FC	1:100
CD11b	M1-70/Rat	FITC	Biolegend	101,206	FC	1:100
F4/80	BM8/Rat	AF700	Biolegend	123,129	FC	1:100
MHC-II	M5-114.15.2/Rat	APC-Cy7	Biolegend	107,602	FC	1:100
Zombie Red	N/A	N/A	Biolegend	423,110	FC	1:500–1000

Cells were washed and fixed for 20 min (eBioscience, 88-842-00, San Diego, CA, USA) at room temperature, protected from light. Immediately following fixation, PBMCs were washed with FACS buffer. This was followed by a series of washes with PBS and permeabi lization. After final washes, samples were resuspended in 300 μL PBS. Data were immediately acquired on a Sony Spectral Analyzer SP6800 or Cytek Aurora 5 L Spectral Analyzer. Each experiment included single color compensation, followed by automatic compensation calculation. Compensation matrices were not altered thereafter. Data were analyzed using FlowJo (Treestar Software; Version 9; Ashland OR, USA), using analysis gates set by FMO (Supplemental Figures S1–S3).

### 2.9. Phagocytosis

To measure phagocytosis, splenocytes and peritoneal macrophages were incubated with 0.5 mL fluorescent latex beads (L3280, Sigma, Saint Louis, MO, USA), then washed twice to remove unbound beads. Bead incubation was conducted simultaneously with flow cytometry antibody staining as described above.

### 2.10. Fluorescence Minus One (FMO) Analysis

Samples stained as described above were analyzed using gates set by fluorescence minus one (FMO). For each panel used, a separate set of samples was prepared in which a single fluorochrome was omitted per sample. After compensation, negative space created by omission of the fluorochrome was used to set positive gates. Set gates were verified using a fully stained sample. This procedure was repeated for each panel. FMO analysis scheme and final gating scheme for each panel are given in Supplemental Figures S1–S3.

### 2.11. Serum Cytokine Analysis

Serum from whole blood was collected, aliquoted and stored as described above. Following the manufacturer’s instructions, serum cytokines were assessed by 13-plex Mouse Inflammation Panel (740446, Biolegend, San Diego, CA, USA). All samples were analyzed in duplicate and technical replicates. Data were acquired on a 5-laster Beckman Coulter Cytoflex LX and analyzed using Biolegend’s Legendplex Analysis software (Version 1.1, San Diego, CA, USA).

### 2.12. Statistical Analysis

Data are presented as mean +SEM. Statistical analysis was performed using Graphpad Prism 8. Differences between groups were determined by t-test (two groups) or one-way ANOVA (more than two groups). When comparing multiple samples via Two-way ANOVA, Sidak’s post hoc test was applied for multiple comparisons. Significant effects shown throughout this study are displayed as effect of genotype (DAT^−/−^ vs. WT).

**Table 3:** Equipment.

Equipment	Supplier	Part Number	Purpose
Centrifuge	Sorvall	ST8	PBMC isolation
Cytometer	BD	Canto II	FC
Spectral Analyzer	Sony	SP6800	FC
Spectral Analyzer	Cytek	Aurora 5 L	FC
Flow cytometer	Beckman	Cytoflex LX	FC, 13-plex ELISA
Microcentrifuge	Fisher	59A	FC

## 3. Results

### 3.1. DAT Deletion Alters the Composition of the Circulating Immune System, Induces Spleen Hypoplasia, and Alters Complete Blood Count (CBC)

**Figure 1:**
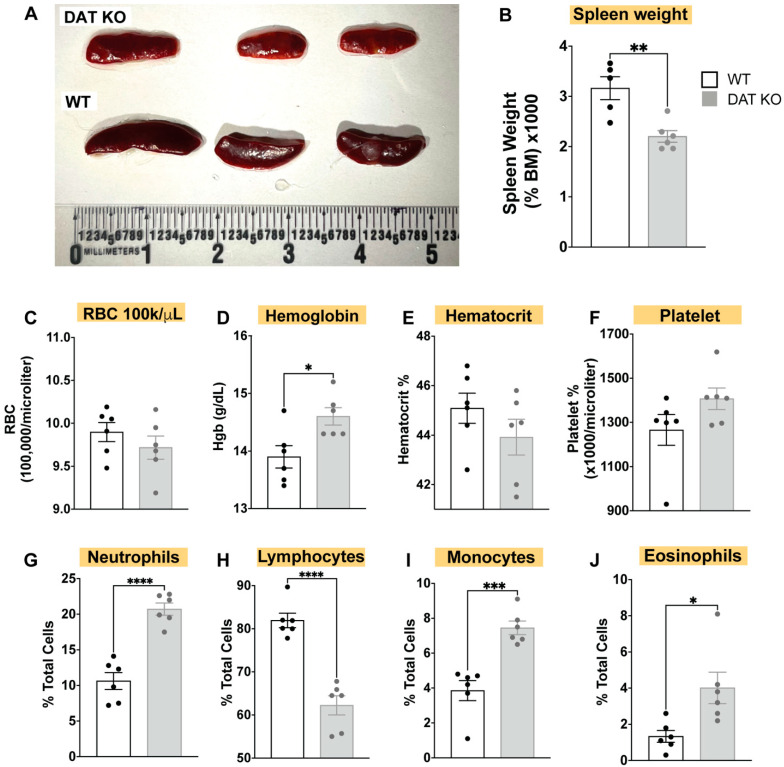
DAT^−/−^ mice exhibit spleen hypoplasia and reduced weight and exhibit altered peripheral immune populations via CBC. (**A**) Qualitative assessment of spleen size and appearance indicates DAT^−/−^ animals show spleen hypoplasia, and splenic pallor, suggesting that DAT^−/−^ mice may exhibit immune dysfunction or anemia, with (**B**) spleen weight taken as a fraction of total body weight confirming reduced spleen size (*n* = 5 WT, *n* = 6 DAT^−/−^). Contrary to qualitative splenic pallor, via complete blood count with differential (CBC-diff) from DAT^−/−^ mice and WT controls we found that (**C**–**F**) DAT^−/−^ mice do not show reduced RBCs, hemoglobin, hematocrit, or platelet density (CBC). However, differential results from CBC-diff shows perturbed WBC populations in DAT^−/−^ mice relative to control, showing increased myeloid lineage cells (neutrophils, monocytes, and eosinophils); (**G**,**I**,**J**) alongside decreased total lymphocytes (**H**). (Unpaired two-tailed *t*-test, with Holm–Sidak correction for multiple comparisons; *n* = 6/group; * *p* < 0.05, ** *p* < 0.01, *** *p* < 0.001, **** *p* < 0.0001).

To investigate the association of DAT to peripheral immune function, we first examined the composition of the circulating immune system at baseline in WT and DAT^−/−^ mice. We first noticed spleen hypoplasia in DAT^−/−^ mice, indicated by a stark change in spleen morphology with DAT^−/−^ spleens appearing paler and smaller (Figure 1A). Indeed, the DAT^−/−^ spleens were smaller than WT controls when normalized to total body weight (Figure 1B). As the spleen is a secondary lymphoid organ and major reservoir for circulating immune cells (spleen also contains 1/3 of the red blood cell population) [27,28], we hypothesized that the changes in spleen size may reflect changes in the relative abundance of circulating immune or red blood cells. We performed a complete blood count (CBC) on the DAT^−/−^ and WT mice and found an increase in DAT^−/−^ hematocrit (*p* < 0.05), but no pronounced differences in blood volume (Figure 1). There was a drastic decrease in the lymphocyte counts in DAT^−/−^ mice compared to WT mice, and an increase in monocyte, neutrophil, and eosinophil counts (*p* < 0.05). Thus, loss of DAT resulted in spleen hypoplasia and corresponding lymphopenia, indicating that the expression of DAT is required for a normal baseline immune profile.

### 3.2. In DAT^−/−^ Mice, Norepinephrine and Dopamine Levels Are Increased in Spleen and Thymus, but Not in Circulating Serum

**Figure 2:**
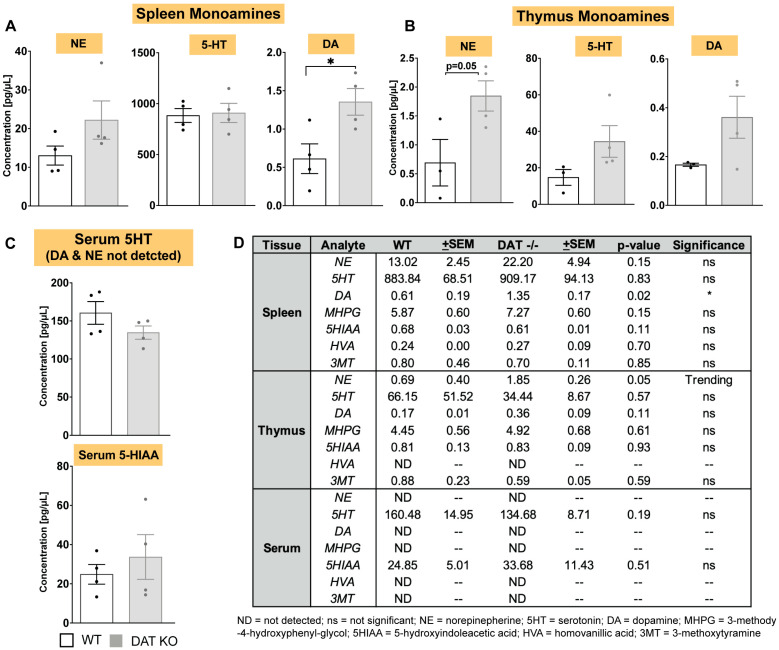
In DAT^−/−^ mice, norepinephrine and dopamine levels are increased in spleen and thymus, but not in circulating serum. Peripheral blood, spleen and thymus were isolated from DAT^−/−^ mice and WT controls. The samples were lysed and assayed via HPLC for monoamines and their metabolites. (**A**) Relative to WT controls, DAT^−/−^ mice exhibit significantly increased splenic dopamine and (**B**) significantly increased thymic norepinephrine, with no change in monoamine metabolites in either spleen or thymus. (**C**) Only serotonin and its metabolite 5-HIAA are detectable in serum of DAT^−/−^ and WT mice; they are not significantly different between the two groups. (**D**) Mean ± SEM values for monoamine levels, their metabolite, *p*-values, and significance for each are shown. (Unpaired two-tailed *t*-test, with Holm–Sidak correction for multiple comparisons; *n* = 3–4/group; * *p* < 0.05).

More than two decades ago Marc Caron lab showed that CNS monoamines are altered in DAT^−/−^ mice [29], but the impact of global DAT deletion on monoamine levels in peripheral tissues and immune organs such spleen and thymus remain unknown. As shown in Figure 1, DAT^−/−^ mice exhibit splenic hypoplasia, indicating the importance of DAT activity in peripheral immunity. Spleen and thymus are two important immune hubs in the periphery, innervated by CNS dopaminergic neurons, and involved in coordination of innate and adaptive immune response [30,31,32]. Therefore, we assessed levels of dopamine, norepinephrine, serotonin, and their metabolites in the spleen, thymus, and serum of DAT^−/−^ and WT mice. In the DAT^−/−^ mice, we found a significant increase in splenic dopamine (*p* < 0.05, *n* = 4) but no significant change in norepinephrine, 5-HT, or their metabolites (Figure 2), whereas in the thymus of DAT^−/−^ mice there is a strong trend (*p* < 0.052, *n* = 4) for increased NE, but no significant change in the other two monoamines or their metabolites (Figure 2). In the serum of DAT^−/−^ mice and WT, only serotonin was detectable that was not different between the two groups (Figure 2). While these data do not determine whether splenic and thymic monoamines are produced within the tissue, derive from descending catecholaminergic innervation, or a combination of these mechanisms, the results support the interpretation that DAT activity regulates peripheral immunity most likely via a tissue-specific mechanism.

### 3.3. Loss of DAT Skews the Myeloid Population to a Pro-Inflammatory Phenotype

Previous work in our lab has demonstrated that DAT activity plays an immunomodulatory role in response to LPS-induced immune stimulation [9]. Specifically, recent reports indicate that myeloid cells may be particularly susceptible to perturbations in dopamine transmission [9,25]. Given the CBC data shown in Figure 1, we conducted more thorough immunophenotyping of myeloid subtypes in WT and DAT^−/−^ mice at baseline. We also investigated how loss of DAT expression may impact the immune response. To this end, we induced systemic endotoxemia [33] via a tail vein injection of lipopolysaccharide (LPS) (2 mg/g), a potent inflammatory stimulus [34,35] in both WT and DAT^−/−^ mice. Four hours after LPS exposure, we harvested blood, serum, peritoneal macrophages, and spleen for detailed immunologic analyses. Consistent with previous reports, an acute dose of LPS (2 ug/g) did not affect immediate survival of WT [9,25] or DAT^−/−^ mice.

**Figure 3:**
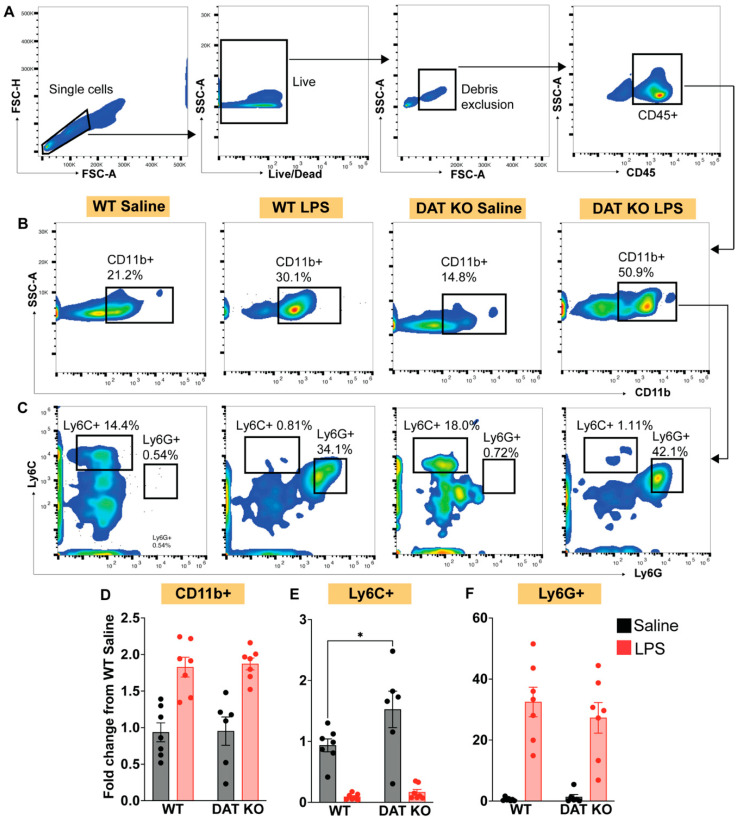
DAT^−/−^ results in exaggerated baseline inflammatory phenotype in peripheral circulating myeloid cells. Adult homozygous DAT^−/−^ animals were treated with saline or tail-vein LPS to mimic acute endotoxemia alongside wildtype littermate controls. (**A**) After gating of single, live cells, free from debris, CD45+ leukocytes were assessed for expression of (**B**) CD11b, which were further assessed for (**C**) Ly6C and Ly6G expression. (**B**) Peripheral blood immunophenotyping reveals that, while DAT^−/−^ and WT animals exhibit similar baseline and LPS-stimulated CD11b+ cells (**D**) and Ly6G+ reactive neutrophils (**F**), we observed an exaggerated baseline inflammatory monocyte phenotype in DAT^−/−^ animals (**E**), suggesting that systemic DAT^−/−^ sets the stage for altered/exaggerated response to endotoxin exposure. (Two-way ANOVA with Sidak’s post hoc test for multiple comparisons; *n* = 6–9 per group. * *p* < 0.05).

We first analyzed circulating myeloid cell expression of CD11b by flow cytometry (Figure 3A,B). Murine monocytes are broadly categorized into two different subclasses based on their expression of either Ly6C or Ly6G [36,37,38], with Ly6C+ monocytes typically reflecting a more pro-inflammatory population [36,39]. Therefore, we gated CD11b+ cells based on Ly6C and Ly6G expression (Figure 3C). DAT^−/−^ mice had similar total numbers of CD11b+ cells at baseline and exhibited a comparable expansion of CD11b+ cells in response to LPS-induced immune stimulation relative to WT controls. However, examination of Ly6C+ and Ly6G+ subpopulations revealed that loss of DAT expression exaggerated baseline inflammatory monocyte phenotype as measured by increased Ly6C+ monocytes (Figure 3E; two-way ANOVA treatment: F(1,23) = 15.77, genotype: F(1,23) = 7.712, and interaction: F(1,23) = 7.942, *p* < 0.05, Sidak’s post hoc test for multiple comparisons). This effect was specific, because, both WT and DAT^−/−^ mice exhibited similar distributions in the Ly6G subpopulation at baseline and in response to LPS-induced immune stimulation (Figure 3F), mirroring the overall expansion in CD11b+ cells, shown in 2D. Thus, DAT deletion results in an exaggerated baseline inflammatory phenotype in peripheral circulating myeloid cells, indicated by increased inflammatory Ly6C+ at baseline.

**Figure 4:**
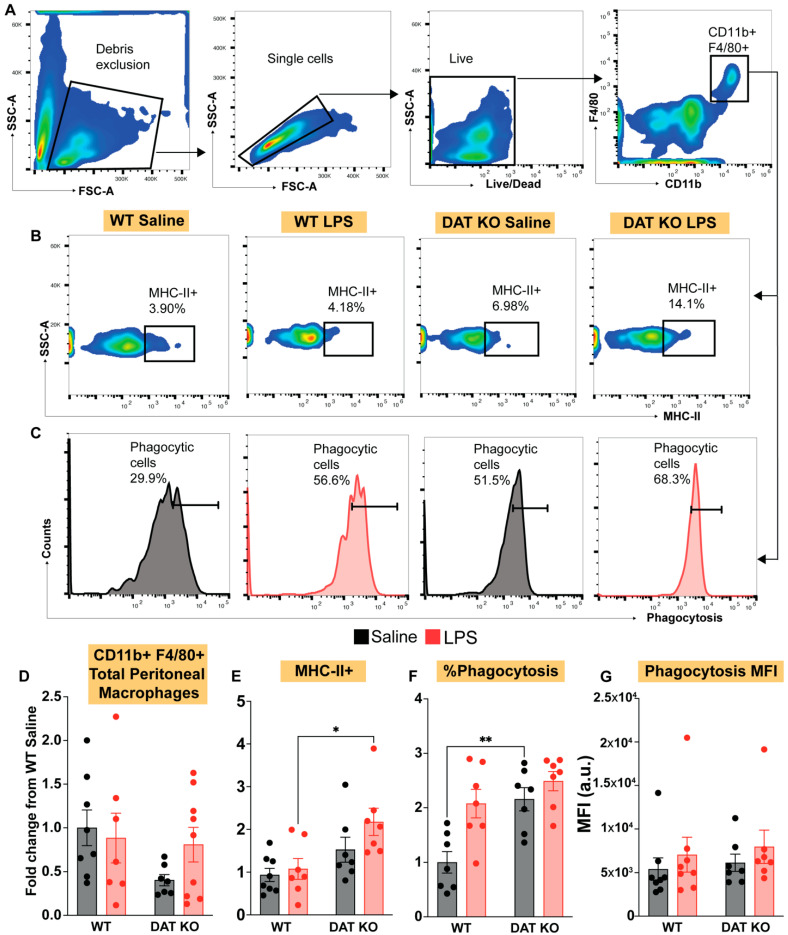
Peritoneal macrophages from DAT^−/−^ animals exhibit increased MHC-II expression and exaggerated phagocytic response. (**A**–**C**) Peritoneal macrophages were harvested and stained for macrophage markers F4/80 and CD11b, MHC-II and assessed for phagocytic capacity. Following LPS-induced immune stimulation, DAT^−/−^ animals exhibit no change in total peritoneal macrophages (**D**) but showed increased MHC-II expression in response to LPS, as compared to WT littermate controls (**B**,**E**). In line with this finding, investigation of phagocytic response to LPS revealed that DAT^−/−^ animals exhibit an exaggerated number of phagocytosing cells both at baseline and following LPS stimulation (**C**,**F**), but with no change in the amount phagocytosed per cell (**G**), indicating a dysregulated response to immune stimulation in the absence of DAT. (Two ANOVA with Sidak’s correction for multiple comparisons; *n* = 6–9 per group. * *p* < 0.05, ** *p* < 0.01).

We next sought to analyze peritoneal myeloid cells, with a particular interest in macrophages, based on previous reports indicating that DAT activity dampen response to immune stimulation [9,25,40]. Taken together with our data from circulating monocytes shown in Figure 3, we hypothesized that macrophages from DAT^−/−^ mice would also exhibit a pronounced pro-inflammatory phenotype. To assess this hypothesis, we harvested peritoneal macrophages 4 h after tail vein injection with either LPS or saline in WT and DAT^−/−^ mice. The peritoneal cavity contains two populations of resident macrophages, which are divided into two main subsets, small and large peritoneal macrophages, based on their size and immune marker expression [41,42]. Large peritoneal macrophages (LPMs) are the predominant subset and have higher expression of F4/80 and CD11b [41,42]. LPMs are responsible for surveying the peritoneal cavity and function as first responders to local insults [41]. We focused on this subset in our analyses (Figure 4A) and examined expression of MHCII and phagocytic capacity in F4/80+/CD11b+ peritoneal macrophages (Figure 4B,C). MHCII is required for antigen presentation by antigen-presenting myeloid cells (macrophages and dendritic cells) [43]. Increases in MHCII expression are correlated with macrophage activation [44,45]. Phagocytosis is one of the primary functions of macrophages [46,47] and is modulated by DAT activity [9]. In agreement with no change in circulating monocytes, there was also no effect of genotype (WT vs. DAT ^−/−^) on the total numbers of CD11b+/F4/80+ peritoneal macrophages in saline or LPS-injected mice. However, loss of DAT enhanced the expansion of MHCII+ macrophages in response to LPS-induced immune stimulation (Figure 4E, two-way ANOVA treatment: F(1,25) = 3.428, genotype: F(1,25) = 11.06, interaction: F(1,25) = 1.003, *p* < 0.01). Additionally, we found that the loss of DAT activity increased the number of phagocytic macrophages at baseline (Figure 4F, two-way ANOVA treatment: F(1,24) = 10.78, genotype: F(1,24) = 13.36, interaction: F(1,24) = 3.018, *p* < 0.01) without increasing the amount of phagocytosis per macrophage (Figure 4G). The increased phagocytosis at basal conditions (*p* < 0.05) and increased expansion of MHCII+ macrophages, increased TNFa (an inflammatory cytokine), and decreased GM-CSF (a cytokine involved in responding to bacteria and autoimmunity) [48] (see data shown in the final figure) following LPS-induced immune stimulation in the DAT^−/−^ mice collectively support the interpretation that loss of DAT expression promotes a pro-inflammatory phenotype in peritoneal macrophages.

**Figure 5:**
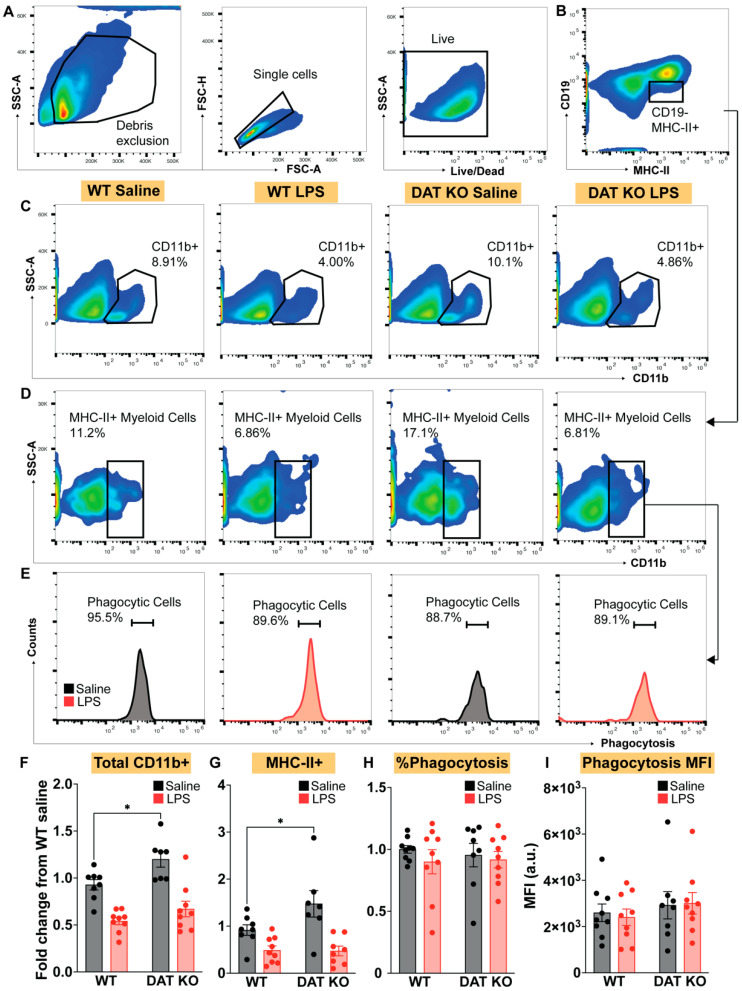
In DAT^−/−^ mice, splenic myeloid cells are increased and show exaggerated MHC-II expression but exhibit no changes in total phagocytosis in response to LPS. (**A**) After excluding debris, cell aggregates and dead cells, total live cells were analyzed for expression of CD11b (**C**). At baseline, DAT^−/−^ animals exhibit increased CD11b+ myeloid cells (**C**,**F**). Cells gated to include only MHC-II expressing CD11b+ myeloid cells (**B**,**D**) revealed increased baseline MHC-II expression in DAT^−/−^ mice compared to WT controls, in the absence of LPS treatment, indicating increased antigen presentation capacity in DAT^−/−^ mice (**G**). However, in contrast to peritoneal macrophages, splenic myeloid cells show no changes in phagocytic response to endotoxin challenge (**E**,**H**), suggesting that presence or absence of DAT differentially impacts immune function in peripheral immune organs in DAT^−/−^ animals. (Two-way ANOVA with Sidak’s correction for multiple comparisons; *n* = 6–9 per group. * *p* < 0.05). (**I**). The relative amount of phagocytosis per macrophage across genotypes and treatment conditions.

Myeloid cells display extraordinary heterogeneity relative to their tissue compartment [49,50]. As a secondary lymphoid organ, the spleen harbors several myeloid populations [51,52]. Given our finding of spleen hypoplasia (Figure 1) and increased splenic dopamine levels in DAT^−/−^ mice (Figure 2), we asked if splenic myeloid populations were affected by loss of DAT (Figure 5A–D). Notably, lack of DAT expression increased both the general CD11b+ populations and the CD11b+/MHCII+ subpopulations in response to LPS-induced immune stimulation (Two-way ANOVA, CD11b—treatment: F(1,29) = 46.43, genotype: F(1,29) = 8.894, interaction: F(1,29) = 1.115; CD11b+ MHCII+—treatment: F(1,28) = 21.62, genotype: F(1,28) = 3.115, interaction: F(1,28) = 3.478) (Figure 5F,G). We also assessed the phagocytic capacity of splenic myeloid cells (Figure 5E). Splenic myeloid cells are efficient phagocytes [53], with a wide variety of phagocytic receptors and mechanisms at their disposal [54,55]. Indeed, our analysis showed that the majority of CD11b+/MHCII+ myeloid cells were phagocytically active in both WT and DAT^−/−^ mice at baseline with no effect of genotype or LPS (Figure 5H). The relative amount of phagocytosis per macrophage was also similar across genotypes and treatment conditions (Figure 5I), suggesting that unlike peritoneal macrophages, either DAT deletion has no effect on splenic myeloid cells’ phagocytosis, or the high phagocytic activity of these cells potentially mask DAT-mediated increase in phagocytosis. Collectively, we found that myeloid cells across body compartments were sensitive to loss of DAT expression. Data in Figure 4 and Figure 5 suggest that a lack of DAT expression was associated with increased markers of antigen presentation and myeloid cell function, along with increased proxies of pro-inflammatory/activated phenotypes. Therefore, although there remain many tissue compartments and myeloid subsets in which to investigate the consequences of DAT deletion, our data strongly support the interpretation that DAT activity serves as an innate biological mechanism to constrain pro-inflammatory myeloid phenotypes.

### 3.4. Loss of DAT Has Divergent Effects on the Circulating Lymphoid Compartment

**Figure 6:**
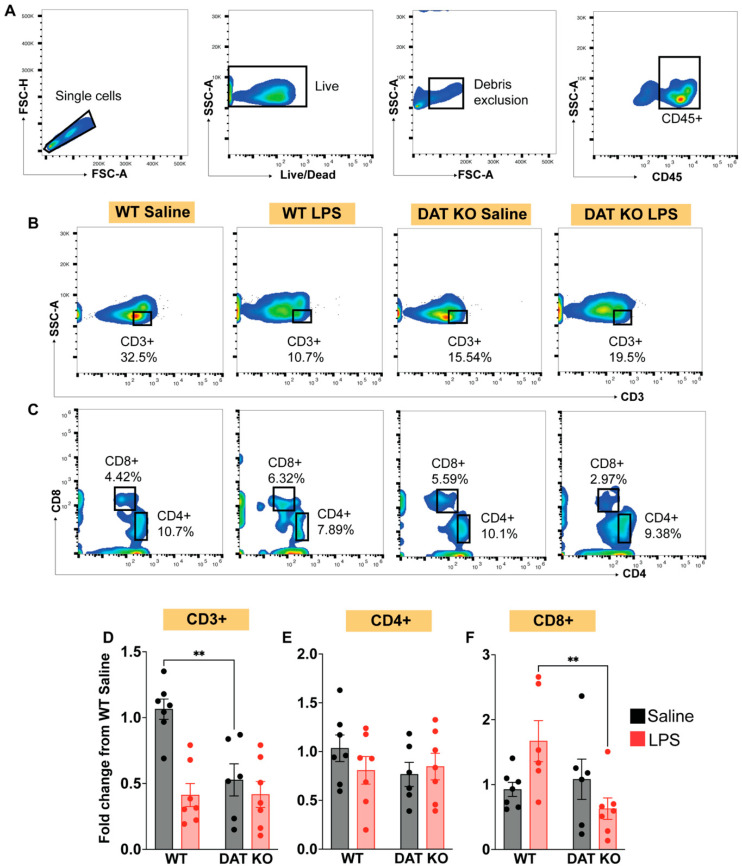
Mice lacking DAT exhibit suppressed T-cell populations both at baseline and following immune stimulation. (**A**) Gating relevant CD45+ leukocytes; (**B**,**C**) assessment of T-cell subsets (CD3+, CD4+, CD8+); (**D**) in WT mice, CD3+ T-cells are reduced following LPS immune stimulation (activation induced cell death, AICD; *p* < 0.05); however, DAT^−/−^ mice show reduced CD3+ cells even at baseline, which is unchanged in response to LPS-induced immune stimulation. (**E**) Further assessments of T-cell subsets show that CD4+ T cells are not significantly altered in any treatment groups regardless of DAT expression or LPS-induced immune stimulation. (**F**) Unlike WT mice, the CD8 T-cells of DAT^−/−^ mice contract following LPS-induced immune stimulation. These results suggest that the adaptive immune system is hobbled in the absence of DAT. (Two-way ANOVA with Sidak’s correction for multiple comparisons; *n* = 6–9 per group. ** *p* < 0.01).

Our data strongly support an immunomodulatory role for DAT in the innate arm of the immune system; however, decreased lymphoid cells (Figure 1H) indicates that the adaptive arm of immune system was also altered in the DAT^−/−^ mice. Therefore, we first investigated CD3+, CD4+, and CD8+ T-cell populations [56] in WT and DAT ^−/−^ mice (Figure 6A–C). In WT mice, immune stimulation reduced the total number of CD3+ T-cells in circulation (*p* < 0.05), representing activation induced cell death (AICD), which is one of the expected responses to systemic LPS immune stimulation [57,58], whereas DAT^−/−^ mice exhibited a baseline contraction of the CD3+ population. In DAT^−/−^ mice after immune stimulation, we did not measure an additional decrease in CD3+ T-cells, which could be due to reduced CD3+ T-cells at baseline, (Figure 6D, two-way ANOVA treatment: F(1,23) = 15.77, genotype: F(1,23) = 7.712, interaction: F(1,23) = 7.942, *p* < 0.01, Sidak’s test for multiple comparisons). However, when we examined the two main subtypes of T-cells, CD4+ and CD8+, we found that the CD4+ T-cell subset was stable across genotypes (WT vs. DAT^−/−^) and treatment conditions (saline vs. LPS) (Figure 6E). In a surprising contrast, in WT mice treated with LPS, CD8+ T-cells expanded, whereas a significant contraction of CD8+ T-cells in DAT^−/−^ mice was observed (two-way ANOVA treatment F(1,23) = 0.4011, genotype: F(1,23) = 3.712, interaction: F(1,23) = 6.787, *p* < 0.01, Sidak’s test for multiple comparisons) (Figure 6F). These data suggest that DAT activity produces distinct and opposing effects in specific immune cell subpopulations within the adaptive immune system. We should note that while CD3+ T-cells decline following LPS-immune stimulation in WT mice, CD8+ T-cells expand, which could be an incongruous observation. However, this decline could be attributed to changes in gamma-delta T-cell populations, which are defined by CD3 (thus the decline in CD3+ T-cells in WT mice following LPS stimulation), but not by CD4 or CD8 expression [59,60]. CD3+ gamma-delta T-cells decline acutely during systemic endotoxemia [60].

**Figure 7:**
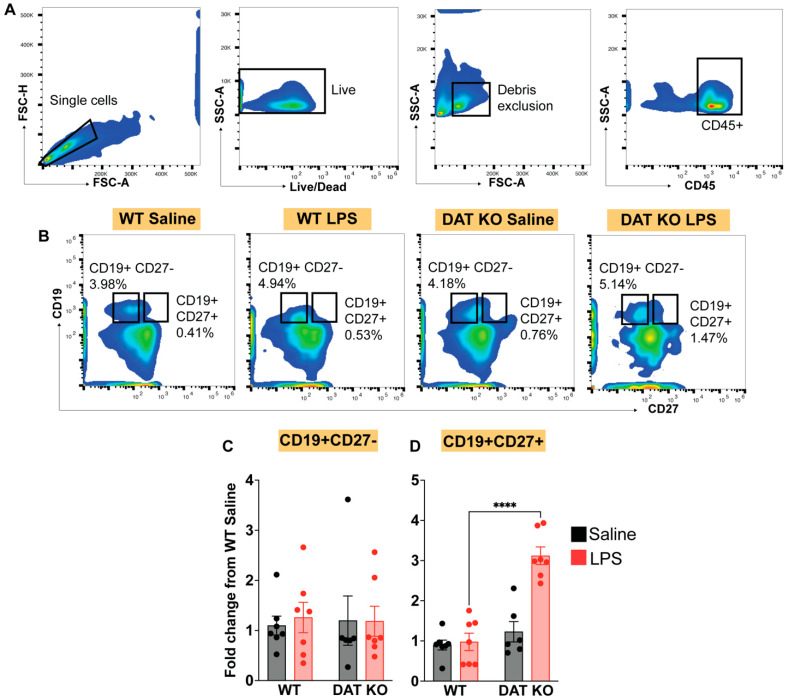
DAT^−/−^ mice show exaggerated memory B cell expansion during systemic endotoxemia, with no change in naïve B-cell populations. (**A**) CD45+ total leukocytes were assessed for B-cell markers CD19 and CD27 (**B**) in each of the four experimental groups with or without acute systemic LPS stimulation. While total naïve B-cells (CD19+ CD27−) were not significantly altered between groups, animals lacking DAT exhibited dramatic expansion of memory B-cells (CD19+ CD27+), which under other circumstances would not be observed with acute systemic endotoxemia (**C**,**D**). These results suggest that systemic DAT is important for canonical B-cell responses to occur; the genetic deletion of DAT predisposes animals to exaggerated expansion of memory B-cells in response to LPS-mediated immune stimulation. (Two-way ANOVA with Sidak’s correction for multiple comparisons; *n* = 6–9 per group; **** *p* < 0.0001).

Within the lymphoid compartment, B cells produce immune responses to pathogens via proliferation, affinity maturation and antibody secretion [61,62]. Therefore, next, we investigated the potential effect of DAT expression on B-cells. We analyzed the circulating B cell populations in WT and DAT^−/−^ animals after saline or LPS tail vein injection. B cells were identified based on expression of CD19, a pan-B cell marker [63,64], and CD27, a marker of memory B-cells [65,66] (Figure 7A,B). While there was not any difference in the total number of B-cells in WT vs. DAT^−/−^ mice (Figure 7C), LPS-induced immune stimulation led to a three-fold expansion of memory B-cells in the DAT^−/−^ mice compared to WT mice (Figure 7D, two-way ANOVA treatment: F(1,23) = 23.06, genotype: F(1,23) = 36.30, interaction: F(1,23) = 19.51, *p* < 0.0001, Sidak’s test for multiple comparisons). This was in stark contrast to what we observed in T-cell populations, where a loss of DAT suppressed CD8 T-cell expansion in response to immune stimulation. These data collectively suggest that the expression of DAT on immune cells has divergent effects on the dynamics of the adaptive immune response.

### 3.5. Memory B-Cell and CD8 T-Cells Express More DAT

**Figure 8:**
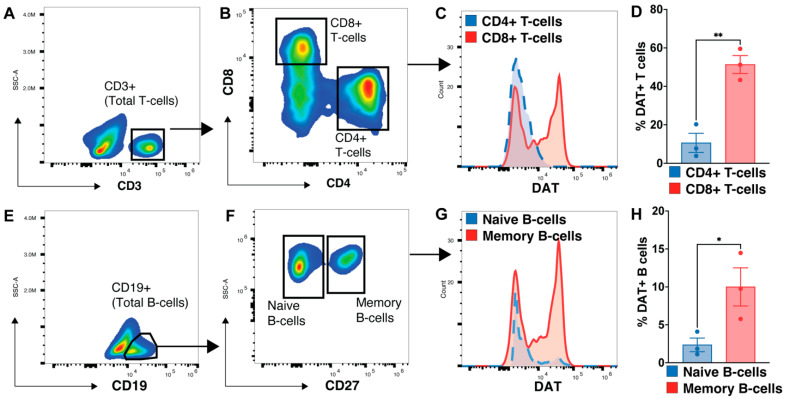
CD8+ T cells and memory B-cells in WT mice express increased DAT relative to other populations, possibly explaining the specific perturbations in B- and T- cell populations of DAT^−/−^ mice. We observed an expansion in memory B-cells but a reduction in CD8+ T-cells in LPS-stimulated DAT^−/−^ mice. Seeking an explanation for these contrasting observations, we conducted a PBMC immunophenotype of these cell populations to assess expression of DAT. (**A**) CD3+ T-cells were analyzed for expression of CD4 and CD8 (**B**). Each T-cell population was then examined for DAT expression (**C**). (**D**) In WT mice, significantly more CD8+ T cells express DAT than CD4+ T cells. (**E**) Similarly, total CD19+ B-cells were separated into naïve and memory B-cells (**F**), with each subset further assessed for DAT expression (**G**). (**H**) Significantly greater numbers of memory B cells express DAT than naïve B-cells. (Two-tailed unpaired *t*-test with Holm–Sidak correction; * *p* < 0.05, ** *p* < 0.01).

Our data suggest that the loss of DAT has contrasting effects on the number of CD8 T-cells and memory B-cells (Figure 6 and Figure 7). Thus, we asked if normal DAT expression (WT mice) on these immune cells exhibits a similar contrasting expression pattern, leading to a divergent response when DAT is absent. Indeed, this observation provides a fundamental role for DAT activity in adaptive immunity in WT mice as well as in conditions where DAT activity is altered. To address this hypothesis, we employed a flow cytometry assay to identify which B- and T-cell subtypes expressed DAT in WT mice. We focused on DAT expression on CD4+ T-cells, CD8+ T-cells, naïve B-cells (CD19+ CD27−), and memory B-cells (CD19+ CD27+) (Figure 8A,B,E,F). Between the two T-cell subtypes, in WT mice, DAT expression was enriched on CD8 T-cells (Figure 8C,D, *p* < 0.01, two-tailed unpaired *t*-test with Holm–Sidak correction). These findings are consistent with data shown in Figure 6, where in DAT^−/−^ mice we observed a reduction in CD8+ T-cells but not CD4+ T-cells, supporting the interpretation that DAT activity is critical to expansion or survival of CD8+ T-cells, potentially describing the attenuated response to immune stimulation in DAT^−/−^ mice shown in the current study, but future in vivo and in vitro studies should examine the effect of DAT on CD8+ T-cell expansion or their viability. Investigation of DAT expression in naïve and memory B-cells of WT mice revealed that DAT is enriched in memory B-cells (CD27+), but not in naïve B-cell (Figure 8G,H, *p* < 0.05, two-tailed unpaired *t*-test with Holm–Sidak correction). These data are consistent with the results shown in Figure 7 that in DAT^−/−^ mice memory B-cells expand in response to LPS. Taken together, these findings suggest that DAT expression has a fundamental immunologic role that is specific to the immunological context, tissue location and cell type.

### 3.6. Loss of DAT Alters the Peripheral Cytokine Profile

**Figure 9:**
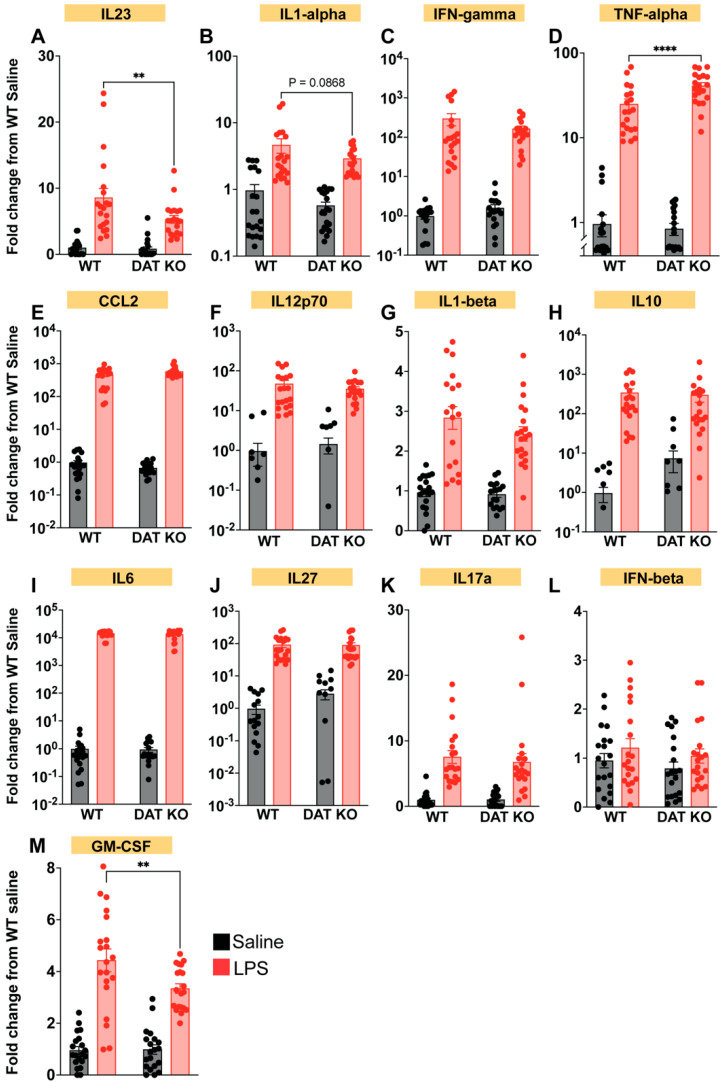
Serum cytokine quantification indicates DAT^−/−^ mice exhibit altered immune response to systemic endotoxemia compared to WT controls. Both DAT^−/−^ and WT animals show serum cytokine changes in response to systemic endotoxemia. However, following immune stimulation, DAT^−/−^ animals show decreased serum IL23 (**A**) and GM-CSF (**M**), and elevated TNF-alpha (**D**), while baseline (saline) cytokine responses are not different between the two groups. No changes were detected in IL1-alpha, IFN-gamma, CCL2, IL12p70, IL1-beta, IL10, IL6, IL27, IL17a, IFN-beta (**B**,**C**,**E**–**L**) These findings are in line with observed changes in some but not all peripheral immune cell populations and functions. (Two-way ANOVA with Sidak’s correction for multiple comparisons; *n* = 8–10 biological replicates, 16–20 technical replicates; ** *p* < 0.01, *** *p* < 0.001, **** *p* < 0.0001).

Thus far, we have examined immune cell subtypes and their activity in blood, peritoneum, and spleen of DAT^−/−^ vs. WT mice at baseline and following LPS-induced immune stimulation, which is a mouse model of systemic endotoxemia. One critical function shared by all immune cells is the secretion of soluble cytokines [67,68,69]. In fact, cytokine secretion and signaling participate in immune responses such as phagocytosis. Since we found that peritoneal macrophages from DAT^−/−^ animals exhibit increased MHC-II expression and exaggerated phagocytic response, we assessed the circulating cytokine profile in WT and DAT^−/−^ mice. The circulating cytokine profile can offer insight into an organism’s general immune state, and serum cytokine profiling is often used in immunophenotyping of various disease states [70,71,72], including systemic endotoxemia [73,74,75]. Given the measured differences in the innate and adaptive immunity of DAT^−/−^ mice at baseline and in response to LPS (Figure 3, Figure 4, Figure 5, Figure 6 and Figure 7), we next asked if DAT expression was important for the cytokine response to LPS-induced immune stimulation. Interestingly, in the absence of immune stimulation, loss of DAT had no effect on most serum cytokines assayed in this study such as IFNg, CCL2, IL12p70, IL1b, IL10, IL6, IL27, IL17a, IFNb. However, loss of DAT reduced the LPS-induced decrease in both IL23 (Figure 9A, two-way ANOVA treatment: F(1,76) = 60.87, genotype: F(1,76) = 4.935, interaction: F(1,76) = 4.080, *p* < 0.01, Sidak’s test for multiple comparisons) and in GM-CSF (Figure 9M, two-way ANOVA, treatment: F(1,76) = 120.7, genotype: F(1,76) = 3.958, interaction: F(1,76) = 4.449, *p* < 0.01 Sidak’s test for multiple comparisons). IL23 is a member of the IL12 superfamily. IL23 is secreted by activated macrophages to enhance the expansion of Th17 cells [76,77]. GM-CSF is secreted by a multitude of activated immune cells [78,79,80] and promotes the survival and differentiation of dendritic cells, granulocytes, and macrophages [80,81,82]. Both IL23 and GM-CSF are involved in pro-inflammatory macrophage polarization [83,84], response to bacterial pathogens [85,86], and crosstalk between innate immune activation and adaptive immunity [87,88]. In WT mice, both IL23 and GM-CSF are increased in response to LPS (Figure 9). In the absence of DAT, reduced GM-CSF suggests that DAT is fundamental to the innate immune responses to LPS. Similarly, in DAT^−/−^ mice reduced IL23 following immune stimulation suggests that innate-to-adaptive crosstalk is impaired in the absence of DAT, supporting our observation of decreased CD8 T-cells (Figure 6), increased memory B-cells (Figure 7) in DAT^−/−^ mice, and increased DAT expression in both subsets (Figure 8). In agreement with these observations, we also found that loss of DAT increased the LPS-induced secretion of TNF, a major pro-inflammatory cytokine released by activated macrophages [89,90,91]. Notably, TNF functions as a negative regulator of the type 1 immune response [92,93], which is consistent with decreased CD8+ T-cells (Figure 5 and Figure 6) and decreased IL23 (Figure 9).

## 4. Discussion, Broader Biological Implications, and Limitations

Here, we show that the dopamine transporter (DAT) on the peripheral immune cells serves as an endogenous immune modulatory mechanism. Using a constitutive global knockout of DAT, DAT^−/−^ [94], we showed that DAT expression is required for a coordinated response to immune stimulation. Specifically, we found that the loss of DAT skewed the myeloid compartment towards a more inflammatory phenotype across multiple tissues such as spleen and peritoneum, enhanced memory B-cell expansion, and suppressed CD8+ T-cell expansion following systemic LPS-induced immune stimulation. The alterations in immune cell composition were accompanied by similar changes in the circulating cytokine profile of DAT^−/−^ mice, with enhanced secretion of pro-inflammatory cytokine TNF and reduction in GM-CSF and IL23 as well as spleen hypoplasia. Thus, DAT expression is important for a wide variety of immune cells and immune functions.

DAT is expressed by dopaminergic neurons of the CNS and by various peripheral immune cells such as monocytes, macrophages, CD8+ T-cells, and memory B-cells [8,9,12,25,40,95]. Since in DAT^−/−^ mice, DAT expression is eliminated on all cell types, there are two potential, non-mutually exclusive mechanisms to explain why loss of DAT results in an altered immune response. The first is that dopamine signaling in the brain can impact the peripheral immune response. In fact, there is already a notion that linking reward processing to immunity confers an evolutionary advantage [96], with one report showing that direct activation of VTA dopamine neurons boosted both the innate and adaptive immune responses to multiple challenges [96,97]. Since loss of DAT would putatively have a similar effect of increasing dopaminergic signaling from VTA dopamine neurons, one potential mechanism explaining our findings is that this dopaminergic neuro-immune modulatory circuit was enhanced by loss of DAT function in the CNS. Chimeric mice with selective knockout of DAT in the CNS can resolve whether CNS DAT is necessary for coordinating the immune response. Even then, there are several unanswered questions remaining about this circuit. Specifically, it is unclear which dopaminergic projections are crucial for the circuit. Areas such as the periventricular nucleus of the hypothalamus and the insular cortex are prime candidates as both have been implicated in the CNS regulation of peripheral immune responses [98,99,100] and receive dopaminergic input [101,102]. Future experiments utilizing anterograde/retrograde tracing combined with activity-dependent labelling (e.g., FosTrap) are suitable approaches to better dissect the precise dopaminergic circuits involved in neuroimmune modulation.

The alternative mechanism is that DAT expression on peripheral immune cells acts in a cell-autonomous fashion to modulate the immune response. Peripheral immune cells engage in autocrine/paracrine dopamine signaling [9], with local concentrations of dopamine modulating immune responses [12]. Indeed, this was the mechanism that our lab discovered for DAT activity on monocyte-derived macrophages [9]. In this scenario, DAT on immune cells would acutely regulate the local dopaminergic tone at the cell membrane, thereby allowing for preferential signaling on high- or low-affinity dopamine receptors. It is important to note that dopamine concentrations in the periphery range from pico- to micromolar levels, meaning it is a physiologically relevant signaling molecule in many tissues and immune compartments [12,103]. It is also worth noting that most immune cells express at least some dopamine receptors [12,104,105,106]. Therefore, it is possible that loss of DAT on immune cells impairs their ability to locally modulate dopamine signaling, leading to dysregulated immune responses. However, due to the wide variety of circulating immune cells and tissue-specific immune cells, addressing this possibility was beyond the scope of this study. Subsequent experiments involving selective knockout of DAT on certain immune cell populations will evaluate the cell-type specific role of immune DAT in peripheral immunity. It is likely that the phenotype we reported in the current study is the combined result of CNS and peripheral immune DAT knockout. Teasing apart these mechanisms will be challenging, but worthwhile because identifying these mechanisms will lead to a significant advancement in the understanding of dopaminergic neuro-immune modulation.

In this study, we investigated how an absence of DAT expression on peripheral immune cells influences peripheral immunity by focusing on major types of circulating immune cells—monocytes, CD4 T-cells, CD8 T-cells, naïve B-cells, and memory B-cells—as well as peritoneal macrophages and CD11b+ MHCII+ splenic myeloid cells. Across these immune cells, our findings consistently supported the hypothesis that DAT expression is required for a coordinated immune response. Our data in circulating monocytes suggest that DAT expression constrains their pro-inflammatory functions, as loss of DAT skewed monocytes towards a more pro-inflammatory Ly6C+ phenotype. Ly6C+ monocytes are chemotactically active and respond rapidly to inflammation or injury [107,108], making them key players in the innate immune response. This agrees with our previous reports showing that DAT activity provides an endogenous anti-inflammatory mechanism for monocyte and macrophage function [9,25]. Importantly, increased Ly6C+ monocytes in DAT^−/−^ mice was present without LPS-induced immune stimulation, suggesting that DAT^−/−^ mice are biased towards a pro-inflammatory phenotype. It is possible that this proinflammatory skewing primes the monocytes in DAT^−/−^ mice toward a more potent inflammatory response upon immune stimulation. This interpretation is supported by a previous report showing that ex vivo stimulation of myeloid cells from DAT^−/−^ mice resulted in increased release of pro-inflammatory cytokines compared to WT mice [109]. Future studies may validate this by conducting a more comprehensive immunophenotyping panel on monocytes before and after immune stimulation in the same animal.

Next, we investigated the consequence of DAT expression on the innate arm of the immune system. We analyzed tissue resident myeloid populations—peritoneal macrophages and splenic CD11b+ MHCII+ myeloid cells. For peritoneal macrophages, we found an upregulation of MHCII following immune stimulation only in the DAT^−/−^ mice (Figure 4). MHCII is an antigen presenting protein, and its upregulation is linked to general immune activation [43]. This finding supports our data on circulating monocytes described above, furthering the narrative that DAT constrains the inflammatory response, and its loss may lead to heightened pro-inflammatory phenotypes. In the spleens of DAT^−/−^ mice, we observed an increase in MHCII+ myeloid cells only at baseline, and this population contracted in response to immune stimulation. A limitation of this study is that we could not account for the diversity of myeloid cells in different tissues. For example, myeloid cells of the spleen are diverse. In contrast, peritoneal macrophages are well defined based on F4/80 and CD11b expression. While our flow cytometry panel captured the effect of DAT deletion in the peritoneum, it is likely that the multiple myeloid cell populations in the spleen, whose responses may be heterogenous and distinct from one another, were not captured by our flow cytometry panel. Multiparameter immune profiling approaches can more accurately reveal the full extent of DAT activity in the splenocytes. Moreover, the contrast between the results in circulating vs. peritoneal vs. splenic myeloid cells highlights the tissue-dependent heterogeneity of myeloid cells, and thus differential influence of DAT on these immune cells.

Investigating the adaptive arm of the immune system, we found a role for DAT regulation of adaptive immune responses (Figure 6, Figure 7 and Figure 8). The consequences of DAT deletion were selective for CD8+ T-cells but not CD4+ T-cells, and memory B-cells but not naïve B-cells, showing specificity for DAT activity on innate vs. adaptive immunity (Figure 6 and Figure 7). This suggests that while DAT can function as an immunomodulator, its immune modulatory properties are cell specific. Our data that CD8+ T-cells and memory B-cells express three-fold more DAT (Figure 8) offer a potential explanation for why these immune cells are preferentially vulnerable to loss of DAT activity. It is possible that these cells utilize DAT to locally modulate dopaminergic tone, or provide a precursor for norepinephrine synthesis, both of which have been shown to regulate peripheral immunity [12,103]. The divergent effects of DAT on immune cells may also reflect dopaminergic signaling pathways on these cell types. For example, low concentrations of dopamine induce T-cell proliferation, with a more dramatic effect on CD8+ T-cells vs. CD4+ T-cells [106,110]. Thus, loss of CD8 T-cell DAT may result in increased dopamine concentrations locally, which would lead to a decrease in CD8 T-cells as we observed in this study (Figure 6). There is one study on dopaminergic regulation of memory B-cell differentiation and proliferation [95] and so this research area requires further investigation. Nevertheless, our data support the conclusion that DAT modulates the adaptive immune response. Building on these findings, future studies are needed to generate an atlas of T-cells and B-cells expressing DAT implicated in a multiplicity of immune states.

In summary, the present study sought to investigate the role of DAT in peripheral immunity in vivo. We found that DAT is a critical player in coordinating the immune response across many immune cell types. Loss of DAT leads to a marked polarization towards a pro-inflammatory state in myeloid cells and shifted the cellular composition of the adaptive arm of the immune system. While there are many outstanding questions that can be addressed in future studies, these results establish a new role for DAT as an immunomodulator.

## Data Availability

All data will be made available upon reasonable request.

## Supplementary Materials

The following supporting information can be downloaded at: https://www.mdpi.com/article/10.3390/cells12020269/s1, Figure S1: Fluorescence minus one analysis strategy for PBMCs. Figure S2: Fluorescence minus one analysis strategy for splenocyte analysis. Figure S3: Fluorescence minus one analysis strategy for analysis of peritoneal macrophages.
