# Supplementary material for: Who Knew? Dopamine Transporter Activity Is Critical in Innate and Adaptive Immune Responses
Source: Cells. 2023 Jan 10;12(2):269. doi: 10.3390/cells12020269 (PMC9857305; doi:10.3390/cells12020269)
Supplement: Supplementary file 1 [file cells-12-00269-s001.zip › cells-2097892-supplementary.pdf]

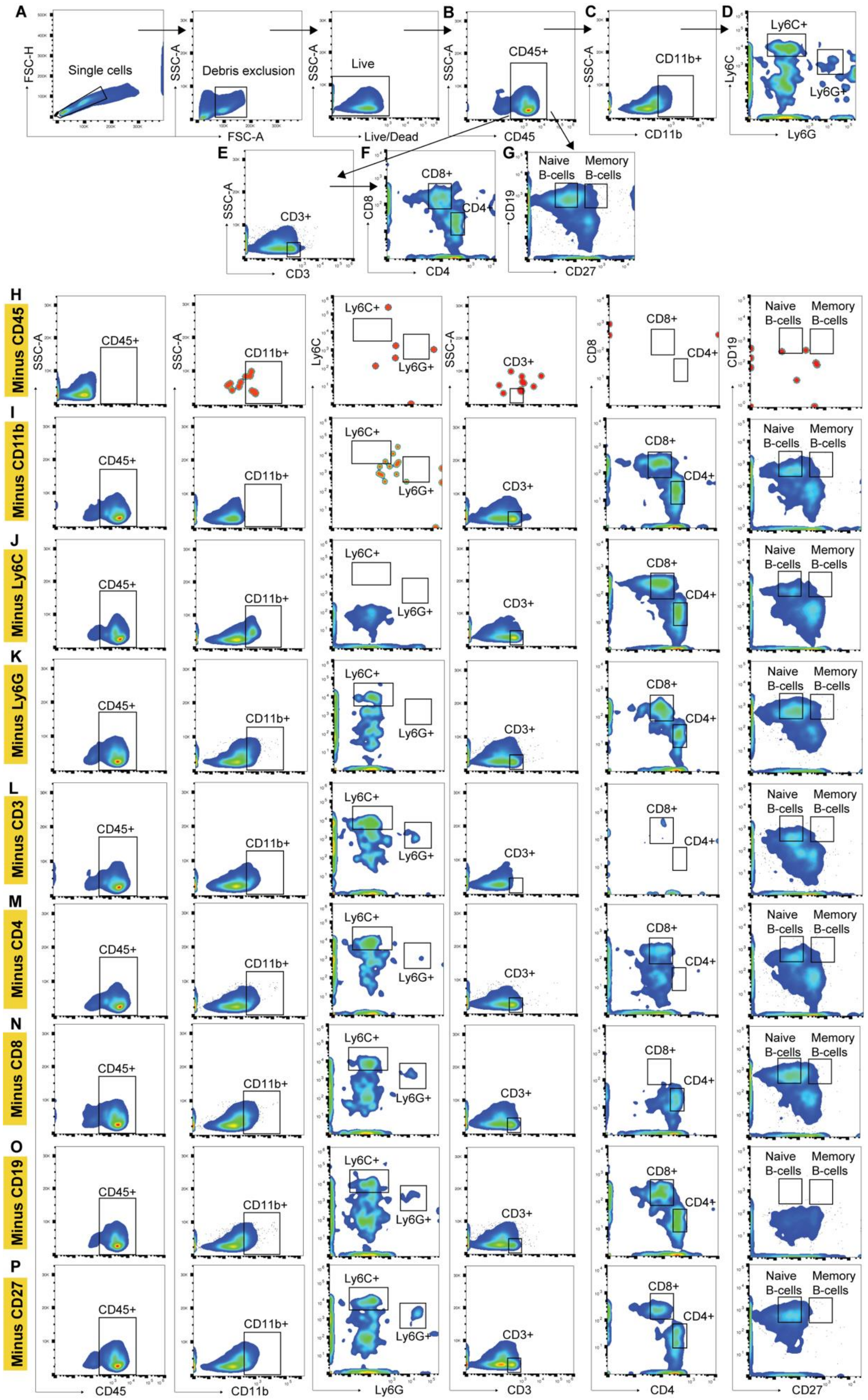
A
B
C
D
E
F
G
H
I
J
K
L
M
N
O
P
FSC-H
FSC-A
Single cells
SSC-A
Debris exclusion
Live
Live/Dead
CD45+
CD45
CD11b+
CD11b
Ly6C+
Ly6G+
Ly6C
Ly6G
CD3+
CD3
CD8+
CD4+
CD8
CD4
Naive B-cells
Memory B-cells
CD19
CD27
Minus CD45
Minus CD11b
Minus Ly6C
Minus Ly6G
Minus CD3
Minus CD4
Minus CD8
Minus CD19
Minus CD27

Supplemental Figure S1. Fluorescence minus one analysis strategy for PBMCs. A) PBMCs were gated for single cells, debris exclusion and live cells, and thereafter analyzed for expression of B) CD45, C) CD11b, D) Ly6C and Ly6G, E) CD3 T-cells, F) CD4 and CD8 T-cells, and G) Naïve and Memory B-cells. Gates were set by fluorescence minus one (FMO) analysis, as shown in H-P, Gating strategy was established by FMO analysis, in which each condition omitted one marker to establish correct gate placement.

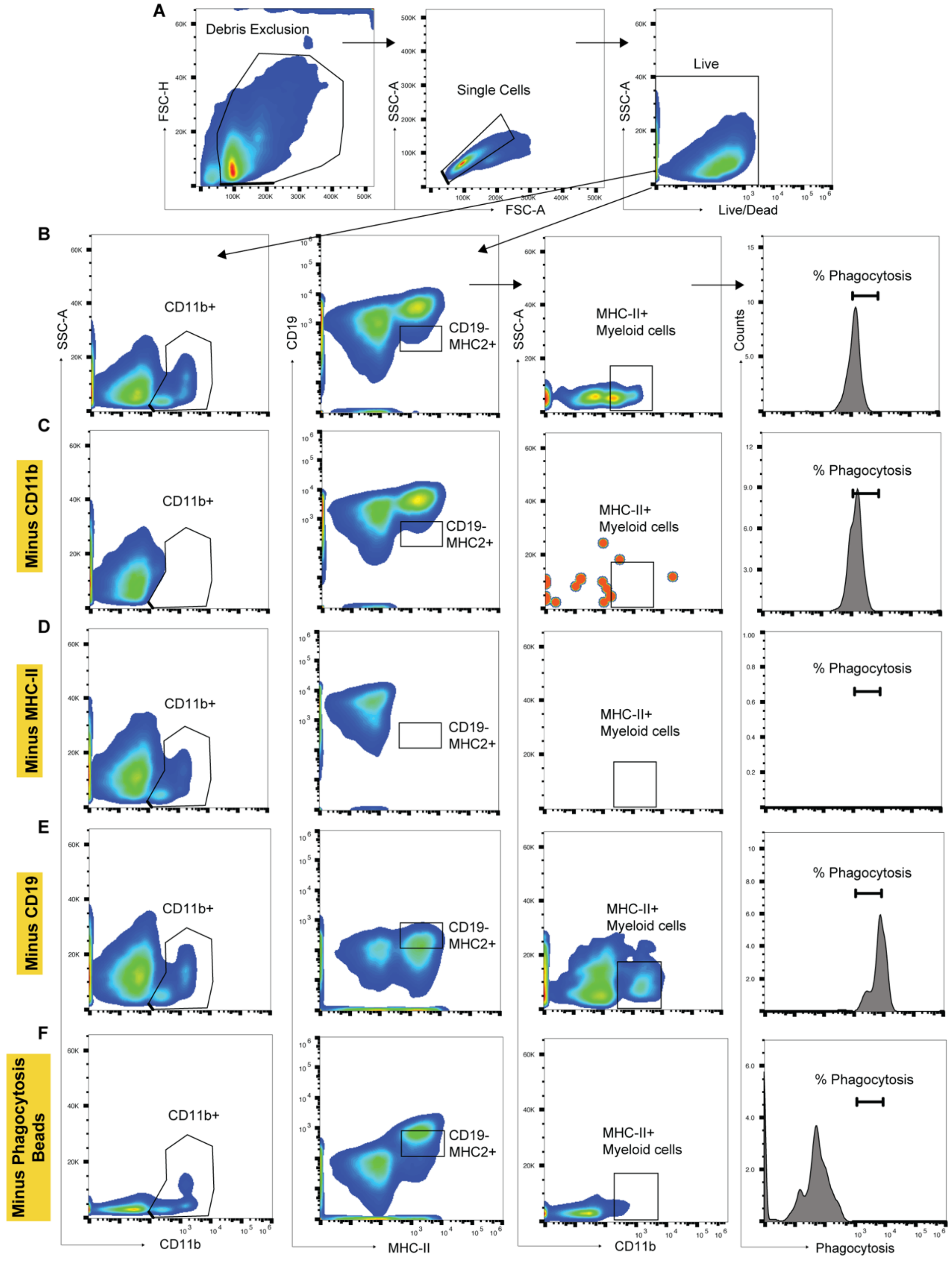

Supplemental Figure S2. Fluorescence minus one analysis strategy for splenocyte analysis. Splenocytes were A) gated for debris exclusion, and single, live cells, and then B) assessed for expression of CD11b, populations of MHC-II+ myeloid cells, and phagocytosis. Gating strategy was established by FMO analysis, in which each condition C-F omitted one marker to establish correct gate placement.

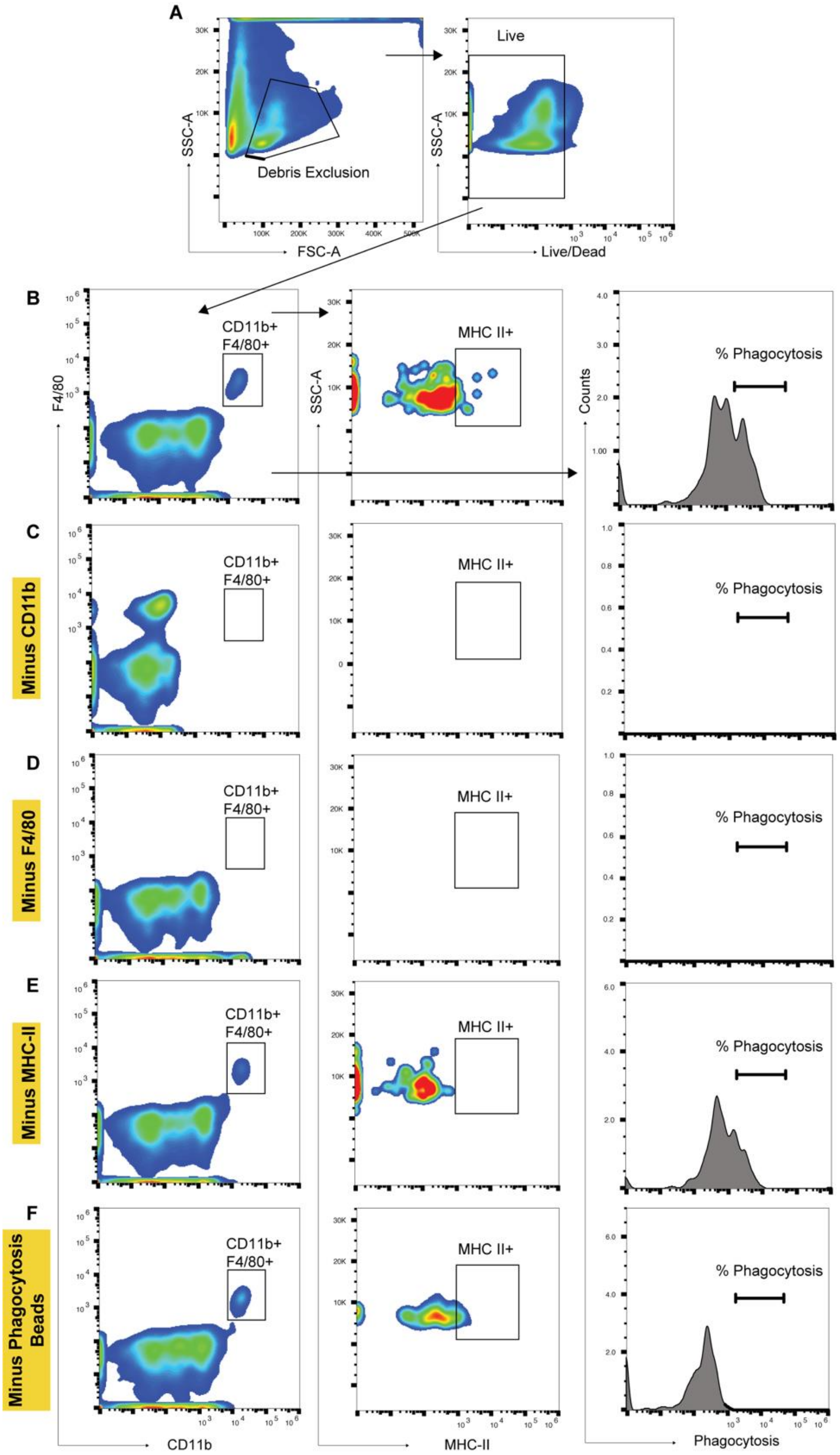

Supplemental Figure S3. Fluorescence minus one analysis strategy for analysis of peritoneal macrophages. A) peritoneal macrophages were analyzed by gating single, live cells and excluding debris,

then assessing these cells for B) expression of CD11b, F4/80, and then assessing those cells for expression of MHC-II and phagocytosis. Gating strategy was established by FMO analysis, in which each condition C-F omitted one marker to establish correct gate placement.
